# Southeast Asian health system challenges and responses to the ‘Andaman Sea refugee crisis’: A qualitative study of health-sector perspectives from Indonesia, Malaysia, Myanmar, and Thailand

**DOI:** 10.1371/journal.pmed.1003143

**Published:** 2020-11-10

**Authors:** Helena Legido-Quigley, Fiona Leh Hoon Chuah, Natasha Howard

**Affiliations:** 1 Saw Swee Hock School of Public Health, National University of Singapore, Singapore; 2 Department of Global Health and Development, London School of Hygiene and Tropical Medicine, London, United Kingdom; International Organization for Migration, SRI LANKA

## Abstract

**Background:**

Southeast Asian countries host signficant numbers of forcibly displaced people. This study was conducted to examine how health systems in Southeast Asia have responded to the health system challenges of forced migration and refugee-related health including the health needs of populations affected by forced displacement; the health systems–level barriers and facilitators in addressing these needs; and the implications of existing health policies relating to forcibly displaced and refugee populations. This study aims to fill in the gap in knowledge by analysing how health systems are organised in Southeast Asia to address the health needs of forcibly displaced people.

**Methods and findings:**

We conducted 30 semistructured interviews with health policy-makers, health service providers, and other experts working in the United Nations (*n* = 6), ministries and public health (*n* = 5), international (*n* = 9) and national civil society (*n* = 7), and academia (*n* = 3) based in Indonesia (*n* = 6), Malaysia (*n* = 10), Myanmar (*n* = 6), and Thailand (*n* = 8). Data were analysed thematically using deductive and inductive coding. Interviewees described the cumulative nature of health risks at each migratory phase. Perceived barriers to addressing migrants’ cumulative health needs were primarily financial, juridico-political, and sociocultural, whereas key facilitators were many health workers’ humanitarian stance and positive national commitment to pursuing universal health coverage (UHC). Across all countries, financial constraints were identified as the main challenges in addressing the comprehensive health needs of refugees and asylum seekers. Participants recommended regional and multisectoral approaches led by national governments, recognising refugee and asylum-seeker contributions, and promoting inclusion and livelihoods. Main study limitations included that we were not able to include migrant voices or those professionals not already interested in migrants.

**Conclusions:**

To our knowledge, this is one of the first qualitative studies to investigate the health concerns and barriers to access among migrants experiencing forced displacement, particularly refugees and asylum seekers, in Southeast Asia. Findings provide practical new insights with implications for informing policy and practice. Overall, sociopolitical inclusion of forcibly displaced populations remains difficult in these four countries despite their significant contributions to host-country economies.

## Abstract 2

In a qualitative study, Helena Legido-Quigley and colleagues investigate how Southeast Asian health systems address refugee and forced migration–related health needs through interviews with healthcare and policy experts and professionals.

## Author summary

**Why was this study done?:**

- Relatively little is known about how health systems in Southeast Asia have responded to the challenges of forced migration and refugee-related health.
- We wanted to identify health system barriers, facilitators, and implications of existing health policies.

**What did the researchers do and find?:**

- We conducted semistructured interviews of policy-makers, experts, healthcare professionals, and academics with professional experience in refugee and asylum-seeker health.
- We used interpretive methods to identify health issues, barriers, and facilitators across the migratory journey.
- Participants recommended regional and multisectoral approaches led by national governments, recognising refugee and asylum-seeker contributions, and promoting inclusion and livelihoods.

**What do these findings mean?:**

- Inclusion of forcibly displaced populations remains difficult in these four countries despite their significant contributions to host-country economies.
- Potential benefits of migrant-inclusive approaches extend beyond preventing disease to improving social equity, livelihood, and population wellness.

## Introduction

In 2018, an unprecedented number of asylum seekers, refugees, and irregular migrants experiencing mass forced displacement due to civil conflict, persecution, human trafficking, and poverty were recorded globally [1]. Worldwide, the sum of individuals forcibly displaced grew to a staggering 70.8 million, with more than a third (25.9 million) crossing international borders in an attempt to seek refuge and asylum [1]. The most globally recognised aspect of this is probably the ongoing ‘European refugee crisis’, which gained significant attention in recent years. Concurrently, a similar crisis occurred in Southeast Asia from 2015 and currently involves irregular maritime migration of refugees and other migrants across the Bay of Bengal and the Andaman Sea [1,2]. In this context, irregular migration, as defined by the UN International Organization for Migration (IOM), refers to movement of persons that takes place outside the laws, regulations, or international agreements governing the entry into or exit from the state of origin, transit, or destination [3].

**Fig 1:**
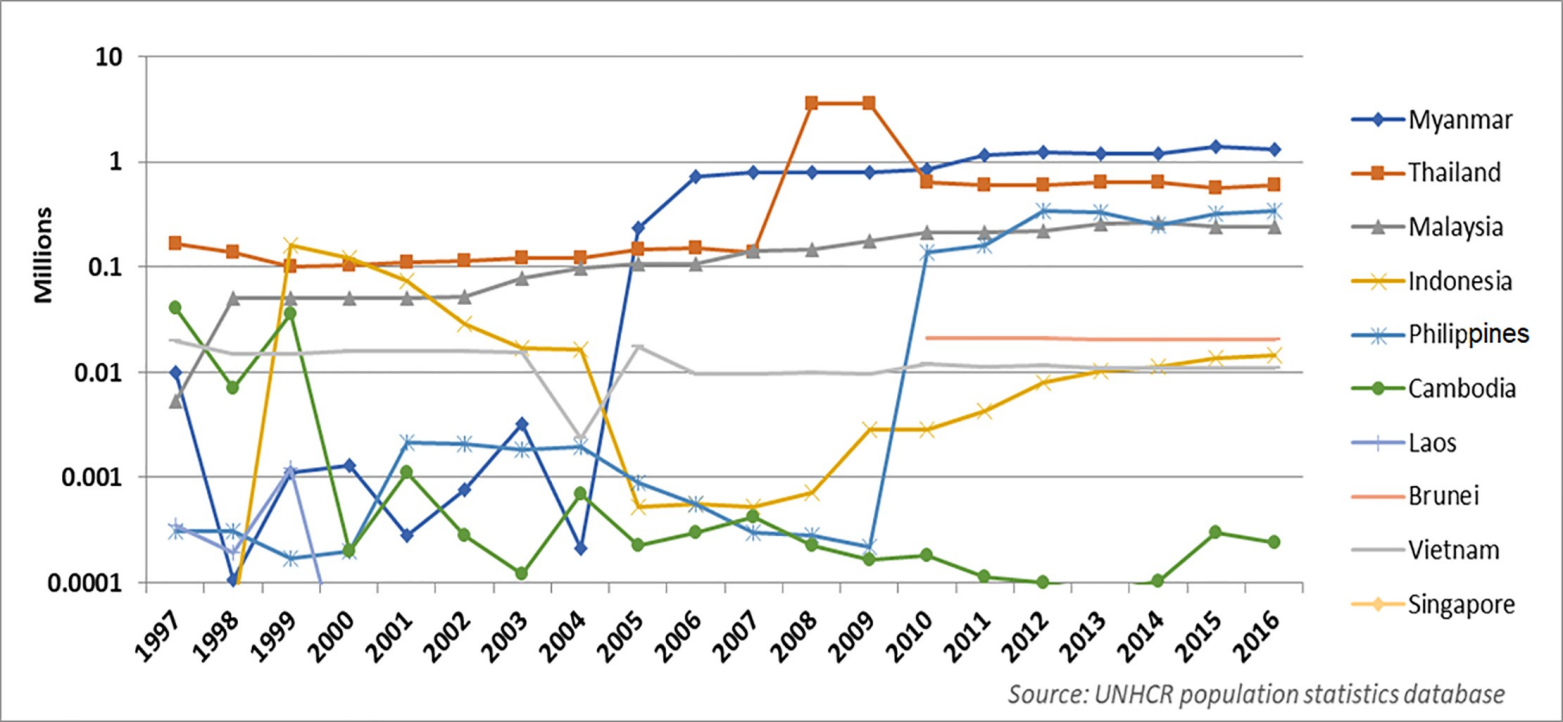
Trends of persons affected by forced displacement in Southeast Asia (1997–2016).

Fig 1 shows Southeast Asia has a complex history of mixed migration that includes populations forcibly displaced by humanitarian crises. For populations affected by forced displacement, the health risks are generally much higher than for economic migrants, as they encounter threats throughout their migration journey and commonly experience protracted detainment and overcrowding, inadequate shelter, food insecurity, and poor water and sanitation [4–6]. When they arrive in countries of transit or temporary asylum, poor access to healthcare services is further compounded by a lack of social networks and assets to effectively navigate the health system. Social determinants of health are compromised for many undocumented migrants and refugees, as their lack of legal status denies them access to work or education [7].

Irregular maritime migration along the migratory route from the Bay of Bengal across the Andaman Sea is cited as one of the most dangerous routes globally with the second highest number of deaths after the Mediterranean Sea routes. From 2012 to 2015, an estimated 170,000 Rohingya refugees and Bangladeshi migrants undertook these dangerous sea crossings to Thailand, Malaysia, and Indonesia [8]. Recently, several key events have highlighted issues affecting such populations and the implications of forced migration on population health. In mid-2015, this irregular maritime migration entered a period of crisis as countries in the region were faced with a humanitarian crisis involving thousands of people on vessels stranded at sea as Rohingya refugees undertook dangerous sea journeys across the Bay of Bengal to escape violence and persecution [2]. An estimated 95,000 Rohingya and Bangladeshi migrants attempted boat crossings to neighbouring countries [9]. The discovery of mass graves at the Thai-Malaysian border led to a series of severe measures on human trafficking [10]. Alongside restrictive policies at sea [11], these maritime routes were disrupted over time [12], and a mass exodus of over 742,000 Rohingya refugees have fled to Bangladesh since August 2017 [13].

Following these incidents, countries in the region acknowledged the need to work collaboratively in devising comprehensive and sustainable approaches to addressing the longstanding issue of irregular migratory movements and refugee influxes in the region [14]. However, much remains unknown about the health needs of these vulnerable populations and the ways in which national and regional frameworks can address health inequities and other social determinants of health.

In Southeast Asia, only 3 of 11 countries are state parties to the 1951 Convention Relating to the Status of Refugees and its 1967 Protocol [5,6]. As most Southeast Asian countries lack formal mechanisms to deal with asylum seekers and refugees, little is known about their level of social inclusion and the challenges that health systems encounter in the financing, coordination, delivery, and capacity to respond to the health challenges of forced migration. As countries continue to seek solutions in addressing forced migration and its implications, this study seeks to address the lack of health policy and systems scholarship in this area to generate further discourse.

The aim of this study was to examine how health systems in the region responded to health challenges of forced migration and refugee-related health from the perspective of healthcare and policy experts and professionals. Objectives were to (1) identify the health needs of populations affected by forced migration; (2) identify health systems–level barriers and facilitators in addressing these needs; and (3) consider implications of existing health policies and potential responses to improve healthcare provision for forcibly displaced and refugee populations.

## Methods

### Study design

We selected a qualitative design, drawing on an interpretive thematic analysis of semistructured interviews with health policy-makers, health service providers, and other experts based in Southeast Asia.

### Study setting

Southeast Asia is a subregion in Asia, consisting of Brunei, Cambodia, East Timor, Indonesia, Laos, Malaysia, Myanmar, Philippines, Singapore, Thailand, and Vietnam [15]. Most of those forcibly displaced in this region are in Indonesia, Malaysia, Myanmar, and Thailand. This study focuses on these four countries’ collective responses to refugee/refugee-like health issues. Table 1 provides a breakdown of numbers of persons of concern to the UN High Commissioner for Refugees (UNHCR) in these countries at the end of 2016 [16–19].

**Table 1:** Study countries ordered by number of persons under UNHCR mandate^1^ by mid-2017.

Groups affected by forced displacement	Myanmar	Thailand	Malaysia	Indonesia
Refugees/refugee-like situations	-	106,447	92,263	7,827
Asylum seekers	-	5,010	56,311	6,578
Stateless persons	925,939	487,741	10,931	-
IDPs	375,016	-	-	-
Returned refugees/IDPs	1,420	-	-	-
Others of concern	-	261	80,000	-
Total	1,302,375	599,459	239,505	14,405

‘-‘ indicates no data available.

^1^ These include refugees, returnees, stateless people, the internally displaced, and asylum seekers.

Abbreviation: IDP, internally displaced person; UNHCR, UN High Commissioner for Refugees.

Myanmar has a long history of civil conflict and large numbers of internally displaced persons (IDPs) and refugees over the decades [20]. In Rakhine State, most IDPs live in camps, more than half of which are women and children [21]. In August 2017, violence led to an exodus of IDPs—mainly Rohingya refugees into neighbouring Bangladesh [13]. Numbers crossing the border to escape the violence have continued to grow, already passing 742,000 since 2017 [13].

Thailand hosts a majority of its refugees and stateless persons from Myanmar in nine Thai government-run ‘temporary shelters’ on the Thai-Myanmar border, with an additional 7,000 refugees and asylum seekers from other countries living in urban areas [22]. Political changes in Myanmar, including the Nationwide Ceasefire Agreement and government elections in 2015 [23], have increased the possibilities of voluntary return for some refugees and stateless persons [22], although this remains challenging [24,25]. On 10 January 2017, Thailand passed a resolution to develop a screening and management system for refugees and undocumented migrants [22]. This was viewed as a positive step towards safeguarding the basic rights, status, and protection of refugees and asylum seekers in the country [26]. Additionally, Thailand’s Compulsory Migrant Health Insurance scheme allows for undocumented migrants, including refugees, to access medical insurance to attend Thai public hospitals [27].

In Malaysia, refugees and asylum seekers live in urban settings alongside host communities and other migrant groups [28]. Malaysia does not possess legal and policy frameworks to address the protection and welfare needs of refugees, asylum seekers, and stateless persons [29]. Refugees recognised by UNHCR Malaysia hold a basic ‘de facto’ status but without any protection from national legislation, which classifies them as illegal or undocumented migrants [30,31]. In the absence of a legal right to remain in Malaysia, refugees do not have access to formal employment and education, and encounter numerous difficulties accessing affordable healthcare [29]. Based on a Memorandum of Understanding between the Malaysian Ministry of Health and UNHCR in 2006, refugees recognised by UNHCR Malaysia are accorded a 50% discount on the foreigners’ rate at public healthcare facilities [32]. However, the cost of medical treatment remains unaffordable, particularly following the 2014 amendment of the 1951 Medical Fees Act involving increasing medical charges for all foreigners by 100% [33].

Indonesia, like its neighbours, is not party to the 1951 Refugee Convention [34]. However, the government has authorised UNHCR to provide protection and identify solutions for the refugee population [35]. In December 2016, the President signed a Presidential Regulation on the Handling of Refugees from Abroad that acknowledges refugees as defined by the 1951 Refugee Convention and stipulates regulations on handling refugees in the country [36]. Refugees in Indonesia are mostly from Afghanistan, Myanmar, and Somalia, and most live in community accommodation [35]. Refugees and asylum seekers under the mandate of UNHCR can access low-cost medical treatment at public healthcare facilities known as ‘Puskesmas’ [37].

Table 2 summarises health system types and access for forced migrants in these four countries.

**Table 2:** Country health systems and status of forced migrants.

Country	Type of health system^a^	Legal status of forced migrants^b^	Healthcare access, costs, risks for forced migrants
Indonesia	• Mixed (decentralised) public/private providers and financing.	• Ratified ICRMW in 2012, but not 1951 Refugee Convention. • Primarily origin/transit rather than destination country.	• Efforts to establish single-payer compulsory national health insurance (JKN).
Malaysia	• Tax-funded, govt-run universal services, growing private sector.	• Not ratified ICRMW or Refugee Convention. • Legal migrants have some protection under domestic laws but undocumented migrants may be arrested, detained, and deported. • Primarily transit/destination country.	• Public universal healthcare is accessible to all legal residents through taxation. • Complex access barriers for undocumented, e.g., identification checks, costs.
Myanmar	• Mixed public/private providers and financing.	• Not ratified ICRMW or Refugee Convention. • Primarily origin country with mix of regular and undocumented emigrants.	• Not applicable
Thailand	• Mixed (decentralised) public/private providers and financing.	• Not ratified ICRMW or Refugee Convention. • Efforts to regularise and provide some protections for migrants, e.g., 2019 Labour Protection in Fisheries Act, Cabinet Resolution 10/01,BE2560. • Primarily transit/destination country.	• Health services accessed through insurance, e.g., universal health coverage scheme for nationals and foreigners with work permits. • Expensive and challenging for undocumented.

^a^As reported by World Health Organization Health System Reviews.

^b^As reported by IOM.

Abbreviations: govt, government; ICRMW, International Convention on the Protection of the Rights of All Migrant Workers and Members of Their Families; IOM, UN International Organization for Migration; JKN, Jaminan Kesehatan Nasional.

### Sampling and data collection

Interviewees were sampled purposively. Those recruited were individuals with professional experience working on refugee health issues or providing healthcare services to refugees and asylum seekers. They included policy-makers, experts, healthcare professionals, and programme personnel from a range of organisations including UN agencies, ministries of health, international and local civil-society organisations (CSOs), and academia. Additional interviewees were then recruited via snowball sampling based on nominations of initial participants. We stopped recruiting when it was collectively agreed that thematic saturation had been achieved—i.e., further interviews were unlikely to provide new ideas or information. A total of 30 interviewees from the region were interviewed, of 30 invited, with none refusing or dropping out. All interviews were conducted in English.

FLHC and HL-Q (both women academic researchers with PhDs) conducted all semistructured interviews, face-to-face or via Skype (see S1 Interview Guide). Potential interviewees were approached via email or phone and given a study information sheet on recruitment and provided written informed consent before further involvement in the study. This included permission for the interview, audio-recording, and anonymous quotation in research outputs. Interviewees were given the option to skip any questions or withdraw at any point. No repeat interviews were conducted. All audio-recordings, transcripts, and research documents were anonymised to protect participant identities and ensure confidentiality. Interviews lasted approximately 60 minutes, were at a place and time selected by interviewees, and were audio recorded. Interviewers used an iterative topic guide to explore health issues and health system challenges and responses from a health-systems perspective. We transcribed recordings verbatim and typed field notes for one interview for which recording was refused.

### Analysis and reflexivity

HL-Q, FLHC, and NH coded transcripts and field notes line-by-line, adopting an interpretive approach to focus on ways in which interviewees understood and made sense of the topics discussed. Data were analysed both deductively (i.e., coding data according to key elements of the interview guide) and inductively (i.e., eliciting new themes or unexpected findings through coding and categorising of data), using QSR Nvivo 12 software to store and manage data. We engaged in an iterative process of developing and reviewing the data through regular discussions to identify a final list of themes and findings. We included deviant cases, whose opinions differed from most interviewees.

All authors have professional experience and interest in refugee and migrant health issues. Two (FLHC, NH) have experience in health and social programmes for refugees in the region. Given our past experiences, self-reflexivity was particularly important throughout the research process so as to avoid the influence of preconceived notions about the research topic. HL-Q’s experience relates instead to the European context, which helped ensure all topics were covered and neutrality maintained throughout data collection and analysis. Authors had no prior relationship with participants prior to study commencement but introduced their research background and interests during the informed consent process. Initial findings were shared with participants who expressed interest or requested it.

### Ethics

Ethical approval was obtained from the National University of Singapore Institutional Review Board. We sought to ensure that findings would not pose any harm to the situation of refugees and asylum seekers in the region, by acquiring feedback and advice from interviewees and experts in the field on the best approach in delivering findings. Our reporting complies with COREQ guidelines.

## Results

Table 3 provides participant characteristics.

**Table 3:** Participant characteristics.

Organisation type	*N*
UN system	6
Ministry of Health/public health facilities	5
International civil-society organisations	9
Local civil-society organisations	7
Academia	3
Total	30
Professional role in relation to refugee work	*N*
Programme manager	12
Programme executive	5
Policy and programmatic work	5
Healthcare professional	5
Academic	4
Total	30
Background	*N*
Clinician	11
Allied health (e.g., pharmacy, psychology, community health)	4
Non-health (e.g., law, economics, operations)	5
Total	30
Country of focus	*N*
Malaysia	10
Thailand	8
Myanmar	6
Indonesia/regional^a^	6
Total	30

^a^Included people based in Indonesia speaking on Indonesia and on the region.

**Fig 2:**
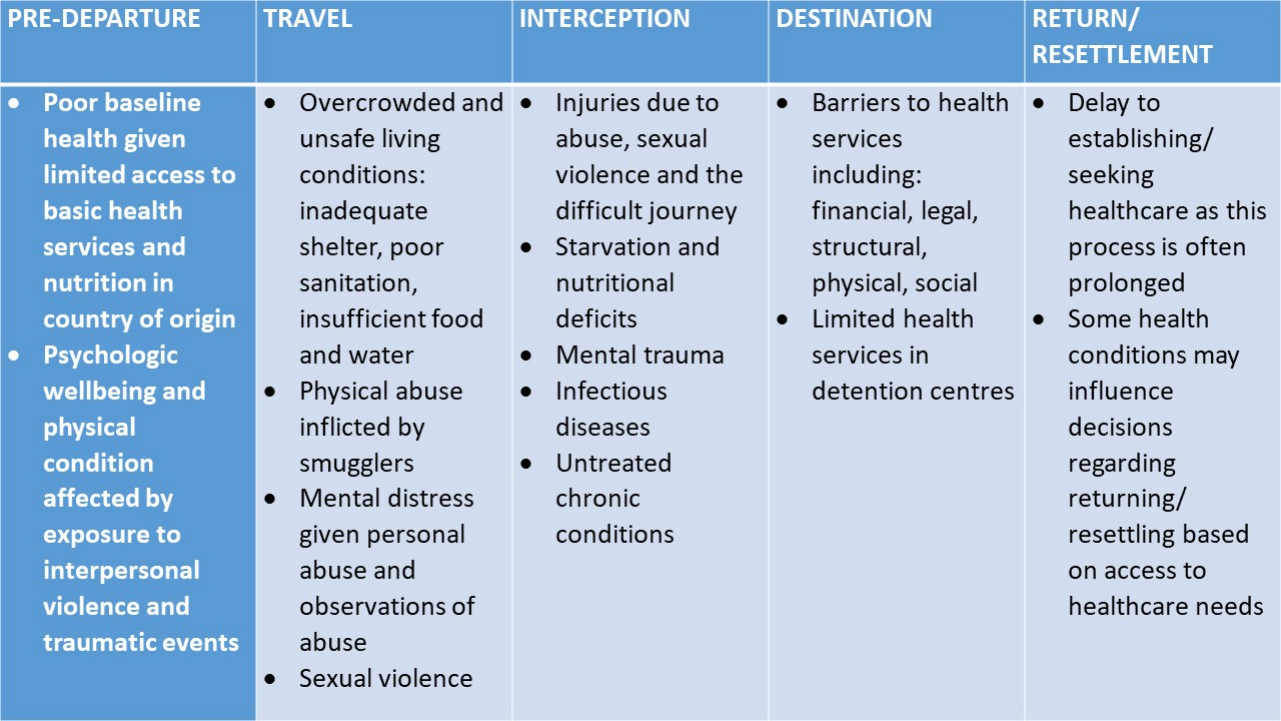
Key health issues reported during the Southeast Asia migratory journey.

Themes are organised under the three objectives of key health issues, health system barriers and enablers, and potential responses and solutions. We used Zimmerman and colleagues’ definitions of displacement and Migratory Process Framework [38], which describes five migration phases spanning the migratory journey (Fig 2), to organise findings under the first theme and highlight the multistaged, cumulative nature of health risks and responses at each phase. The second theme is formed by six subthemes representing both barriers and facilitators to addressing forced migration. These include financial, juridico-political, and sociocultural barriers and the role of health workers and countries’ level of commitment to pursuing universal health coverage (UHC) as key facilitators. The third theme focuses on proposed strategies and solutions as reported by interviewees. Those reported most frequently included the need to find regional solutions, strengthen health systems within the UHC agenda, promote the social determinants of health, and promote social inclusion.

### Health issues across the migratory journey

#### Predeparture

Interviewees described migrants’ poor access to nutrition and basic services in their country of origin, often leading to health issues prior to departure or resulting in worsening health during travel or on arrival. Interviewees reported that refugees were often exposed to violence and sexual assault, leading to physical and mental trauma. Particularly detrimental were predeparture stays in human trafficking camps, which many refugees experienced.

In many cases, these were just places in the jungle where the smugglers had set up cages, almost—wooden cages, some were found, literally cages sometimes—where people were kept in similarly terrible conditions. No food or very little food. People again being shot or beaten to death if they tried to escape or anything of that sort. Almost for sure there was sexual assault. It’s kind of underreported; women don’t speak about it very often but it’s almost certain that there was quite a lot of it and you know that there were hundreds of people who died this way. We don’t have a good estimate because it’s hard to get an accurate count. (I17)

#### Travel

Refugees were often transported in overcrowded vessels, after sometimes extended periods in jungle-based smuggling camps with inadequate shelter, sanitation, and food.

When they leave the country, back home their condition might not be ideal, so there’s already some history behind it. During the movement, it’s difficult to maintain your hygiene and health. And then when you arrive, it’s a totally new environment. It’s a shock basically for anybody. (I05)

Interviewees reported that many refugees witnessed or experienced physical violence and abuse onboard. The next quote highlights the conditions people experienced during travel:

The conditions on-board were pretty terrible. I mean, as bad as you can imagine, basically. People were packed, couldn’t move, weren’t allowed to move, fed very little—usually just a bowl of rice, a cup of water a day. Sanitary conditions were kind of non-existent. In some cases,… people were being killed on-board, probably as a way to keep discipline on-board so the people who tried to kind of start trouble or ask for more food or ask to move around would be beaten and sometimes fatally… (I17)

Rates of trauma, physical illness, and deaths were reportedly high.

The rate of death on those journeys, based on our interviews, seemed to be about 1.1–1.2% which, at the time, was much deadlier than any other crossing in the world. It was about three times deadlier than the Mediterranean at the time… (I17)

#### Interception

Interviewees reported that many refugees experienced immediate health needs on arrival in host countries, particularly skin and respiratory infections due to predeparture and onboard crowding, injuries and trauma due to violence and abuse, and malnutrition due to insufficient food and water.

We saw some physical injuries, some skin infections, malnutrition, probably some psychosocial problems, psychological problems. And in the latest screening, we found that they have positive cases of leprosy and TB [tuberculosis], but the numbers are very small. (I14)

Those who were detained on arrival, e.g., in Malaysia, reportedly experienced unmet needs including lack of mental health or noncommunicable-disease treatment and poor detention conditions.

… Skin, TB, this kind of thing, because the [detention centre] is quite small and they have to fit so many people inside. So one got the disease, all people got the same disease. (I05)

However, one interviewee suggested that refugees were generally relatively healthy despite the traumatic conditions they faced.

… for the most part, I don’t remember hearing about a lot of health concerns, to be honest. I mean, there was the initial… I think, when people first landed, people were malnourished. There would a couple of people who died from illness.... And then, there were people who had died on route. (I17)

#### Destination

Interviewees described ongoing unmet needs as mental health, noncommunicable diseases, and detention conditions (as some countries kept refugees who had experienced forced displacement in detention for extended periods).

Mostly it's flu, gastric pain, the rashes; as usual, the rashes. But a lot of them are also I suppose you can call them mentally disturbed kind of things. They're so anxious, so under pressure from the authorities.... Oh, everybody's itchy; they've got rashes. Scabies is now very common there, scabies and fungus and—because of the lack of hygiene. And everybody's coughing and runny nose. But you get some injuries, some of them from beatings from the guards and some among themselves, when they fight. And also there's the usual—they have gastric pain, they cannot sleep, they have headaches. The headaches, I think—the headache is very common among them. Headaches, I think it's partly because of no ventilation. (I06)

Many interviewees noted that Rohingya refugees were slow to attend health facilities. As the next quote illustrates, it was reported that they only attended health facilities for life-threatening conditions:

Most of the time that we dealt with these migrants, there’s a lot of issue, especially the first impression will be why they come in so late. Usually, they come in, just for example, if you’ve got flu, fever, they will just sort of handle it at home, traditional way. They only come here when there’s a severe, life-threatening, and probably fatal condition—like, if they fell from twenty stories and they cannot treat it at home. (I01)

Interviewees suggested that this could be due to a range of issues, from lack of trust in medical services, to financial and other access barriers, to fear of being identified and detained by authorities. Noting that these concerns were sometimes valid, interviewees acknowledged the ethical issues, with some questioning the value of detention for forced refugees.

#### Resettlement

Returning to their country of origin was not an option for many of these refugees, but interviewees said resettlement required considerable time and depended on many factors over which refugees had little control. Thus, many refugees remained in limbo for protracted periods in the hope of eventual resettlement and during this time increasingly experienced host-country health issues.

So in a certain way, they already have adapted to the [host] country lifestyle, which somehow Malaysia is considered one of the highest countries of risk of chronic disease, which is diabetes and hypertension. (I07)

Some interviewees noted that migrant families’ poor nutrition after resettlement could still be related to income. The next quote highlights that unhealthy diets and too many carbohydrates could be due to cost:

… over-nutrition doesn’t mean that you have too much to eat. It’s also selection of wrong foods.... [Migrants] are eating too much carbohydrates, but you can understand because carbohydrates are cheap. (I04)

While acknowledging refugees’ tremendous resilience, interviewees described other health and social issues that appeared related to the lasting consequences of unresolved complex trauma. The next quote describes the two complementary schools of thought regarding migrant resilience:

I mean, there are two schools of thought, right? One is that they’re very resilient. They can bounce back and they’ve gone through adversities which we can’t even imagine going through.... But resilience doesn’t mean that they’re not affected and that the effect is not long-term. I think that’s the other school of thought that complements our understanding of resilience because sometimes we think they’re resilient; they’re fine just because they look all right and they’re functioning, but that’s not true. Resilience does not mean that they will not benefit from support and health. (I08)

### Barriers and facilitators to health services access for refugees affected by forced displacement

Interviewees explained that refugees affected by forced displacement continued to experience challenges to health services access even when they had been in their host countries for longer periods, including financial, juridico-political, and sociocultural barriers. Financial barriers described by interviewees included poverty, lack of livelihoods, and travel costs and related challenges. Juridico-political barriers included lack of legal, political, and structural support and exclusionary health and social policies. Sociocultural barriers included poor health literacy, lack of familial and social networks to help navigate health systems, and exclusionary biases and social norms.

#### Financial barriers

Interviewees identified insufficient financing of healthcare for refugees as the most significant barrier in all countries, including either directly by refugees, through UNHCR for refugees and asylum seekers, or through specific government or private insurance schemes. As national health budgets were already strained and healthcare was perceived by many as a commodity to be prioritised for citizens, interviewees indicated there was often limited political will to extend healthcare subsidies and services to foreigners, including refugees and asylum seekers.

… it’s all donor-dependent and so that is important because the donor support has somehow reduced and we fear that the donors may, at one point, perhaps sooner than later at one point sort of stop support. (I20)

As nongovernmental migrant health funding was largely project-driven, coherence and sustainability were ongoing concerns among interviewees.

A lot of NGOs [nongovernmental organisations] provides health insurance for refugees or migrant workers, but it’s not really sustainable. It’s a project basis. But we need to think something more. But it’s about the burden sharing. (I18)

Interviewees reported that inadequate governance structures and coordination mechanisms among providers led to fragmented care for refugees and other vulnerable refugees.

There are people willing to provide the service but I think money is the issue. Funding is the issue. I’m not sure if UN is really cutting budget and it’s affecting the mental healthcare for the refugees. But, yes, certainly not enough.... I mean, NGOs are trying to step up but they are very limited in their resources. (I08)

#### Juridico-political barriers

In some countries, migrant healthcare funding challenges were exacerbated by exclusionary policies. Migrant-exclusionary policies, such as limiting their access to essential medicines, were reported in some countries. For example, Malaysia began charging all foreigners considerably higher rates than citizens for healthcare.

The big thing happening… is the increasing cost—increasing cost for migrants. They don’t care what type of migrant you are. Basically, they sort of triple the prices and you pay full amount without any subsidy or funding from our national government. Basically, this per se itself I guess is a bit sad and basically against human rights because they might not have basic healthcare access based on reduced accessibility and affordability. I think it’s a regressive move that, because of limited access, you can actually increase public health sort of problems. (I01)

Most countries distinguished between formal refugees, as designated by UNHCR, and other displaced people who lacked appropriate documents.

If you are labelled as refugee, you are entitled to these kind of services because you are registered as refugee and you are put under the name of refugees. You are entitled to these services. But the same people, they are not in the same group, but they are in the same area, he’s not labelled as refugee, right? But if he needs the similar services like his neighbours, then it makes problems. And then it’s a vicious cycle. (I27)

In some countries, refugees could face detention for seeking healthcare.

… very common in the government hospital is most of these migrants, refugees, or asylum-seekers, they don’t have proper documentation so some of them actually got caught before they’re discharged. They get sent to the detention centre which is quite sad because, from the medical point of view, health staff are supposed to provide medical treatment without taking into consideration criminality or legality. (I01)

Some interviewees suggested that exclusionary policies were deliberately hostile to reduce refugee numbers. The next quote provides an example of multiple responses on exclusionary policies, but at the same time highlighting that restricting policies can cause health problems for host populations:

I think it's their way to get them out, or not to let any more [refugees] in. But the people are coming in, not many, but still coming in. From the very start, [government] never wanted the refugees here, they never wanted UNHCR here, so now I think they are making it more difficult. At one time, they were saying about TB, that they had to pay for the TB medication. Then they realised that if the local population was going to be at risk, because if they don't treat their TB, we will get the TB, so they stopped that. Only for second line drugs, they have to pay. (I06)

#### Sociocultural barriers

Many interviewees described how social biases shaped the political realities that influenced both health funding requests and programming for displaced populations, particularly by CSOs.

[The] relationship between local CSOs and the international community has been pretty poor and been dominated by this anti-international sentiment, which is created by the fact that basically about 90–95% of the funding in previous years has gone to displaced populations. 90–95% of displaced populations are Muslims, so there’s a perception among Rakhine community that the international community is favourable to Muslims.... We’re now… doing more sort of equitable programming, so looking at programs that you know—so 50% Rakhine/50% Muslim, 70% Rakhine/30% Muslim, to try and avoid that perception… (I24)

Several interviewees highlighted that some governments were feeding popular biases against foreigners. The next quote highlights how government used negative rhetoric about illegal migrants when referring to refugees and asylum seekers:

… in Indonesia, apart from maybe some Rohingyas who have some sympathy from the Indonesian population, but otherwise Indonesian population is not sympathetic to asylum-seekers or refugees. They don’t really know the concept, and the government is having also, of course, rhetoric of illegal migrants when they speak about refugees or asylum-seekers. It doesn’t help the population to understand what’s at stake. So for the medical staff as well, they will have various attitudes. People that will be sympathetic and try to help, and people that will not try to help. (I19)

Although most interviewees praised compassionate responses from overworked health workers, some described communication and other difficulties between refugees and service providers.

Sadly speaking, we still see this in the hospital and I do have patients or refugees in my clinic telling me that, “No, they don’t treat me as a human being sometimes because of my look, because of my birthplace.... it may be due to a language barrier. It may be due to you’re not going to pay the bill so why am I going to treat you better? Or just because you are a foreigner, you are occupying one of my beds; now, my auntie cannot be admitted.” (I01)

The main sociocultural facilitator appeared to be the humanitarian stance of many health workers and NGO staff, who wanted to actively provide health services to refugees and migrants in refugee-like situations. The primary juridico-political facilitator appeared to be a strong governmental commitment to UHC, particularly those explicitly incorporating migrant-inclusive policies.

#### Health workers as facilitators

Many described public and civil-society health workers as generally committed to humanitarian and medical principles of providing treatment to all who needed care, regardless of legal and social status.

[Payment is] up to the generosity of each hospital. And from my experience, Thai professionals—Thai medical professionals are not that harsh. So, okay, they may ask you to pay, but if you can't pay, the debt is incurred by that facility. (I15)

In some cases, health workers took direct and potentially controversial approaches to universalising access, as described by one interviewee.

We are very good at doing illegal things to solve the problem. So, we sell the health card to these illegal migrants but selling health cards to these migrants are voluntary. They themselves know that they are illegal so they are afraid that, when they come and show themselves, they’ll be arrested or whatever. But, after a few years, they start to know that we don’t care about their nationality. Once they buy it, we sell, we get money. Or else, when they come to the hospital, they have to pay. (I15)

#### UHC

As UHC requires that migrants also have access to health services, some interviewees suggested that countries should enable all migrants to integrate into the workforce and contribute more taxes so everyone could benefit from better services.

[Migrants] are working in Malaysia. If they can get a formal sector, they can be taxed.... The tax will contribute back to the community. It can be citizen. It can be Malaysian, non-Malaysian. It can be whoever. And then, I think, in the long term, it can be used as a model to actually provide a proper universal healthcare access if you treat everyone the same. (I01)

Countries' level of commitment to UHC and interest in pursuing UHC principles had a significant impact on their support of refugees and other migrants.

… so the government is now pushing for that universal coverage and because of that they listen… they are more opened up. They listen to many other different stakeholders. Um, so they have included many NGOs, GOs [governmental organisations], and ethnic health organizations. (I28)

### Potential responses and suggested solutions

We asked interviewees to reflect on potential responses and suggested solutions to improve the situation of those affected by forced displacement in Southeast Asia. We summarised interviewee accounts into five subthemes: fostering regional cooperation, proposing a different perspective, strengthening health systems within the UHC agenda, addressing the social determinants of health, and promoting social inclusion through livelihood strategies.

#### Regional cooperation

Interviewees described regional cooperation and responsibility sharing as essential for developing solutions, because of the cross-border nature of migratory movements and the fact that policy development was reported as reactive at national level. Several highlighted the need to regularise the movement of people in the region and have a common response to the current refugee crisis. Most, as the next quote illustrates, considered the Association of Southeast Asian Nations (ASEAN) the most appropriate platform for this:

Definitely. It's within these countries that the movement of migrants are occurring, and so it's only in their own interest to actually look at a more regularised movement of people in the region, and to address that this sort of crisis when it happens, and to address also the source of why it is happening with the relevant countries, who are the source of—the refugee producing countries within the region.... So ASEAN is the prefect platform for actually dealing with this, not just from the political level, but also in all the response levels, from health and other sectors, to come in on this. (I09)

Interviewees also described the importance of using ASEAN to facilitate discourse on resolving the root causes of forced migration and showing “that they are really serious about tackling this problem” (I14). The next quote illustrates the need to address push factors and the root causes of forced migration:

The issue is a push factor. It's not the host country. We are the victims, isn't it? Malaysia, Thailand, Indonesia. I mean, you have to tackle the root case.... Until you settle this root cause, the push factor, if there's no push factor, they will not leave. If they don't leave, there should not be any refugee issues. (I14)

Interviewees highlighted that all actors had a role in finding solutions, including UN agencies, health and other relevant ministries, international and national NGOs and community-based organisations and pressure/interest groups, media, private-sector organisations, health facilities and health workers, funding agencies, and governments of origin, interception, and resettlement countries. However, interviewees agreed that governments in the region had enduring dominance over other actors in the formulation and evolution of policies. Overall, they suggested that all key actors had to come together in the same platform to find practical solutions:

Basically, I think the humanitarian organizations, the government, the NGOs, the civil societies all need to come to the same platform for all these migrant issues. (I01)

#### Need for a change in perspective

Interviewees expressed the need for a paradigm shift in dealing with refugees and asylum seekers, by first promoting ongoing dialogue to facilitate understanding of their situations. As one highlighted:

The ongoing dialogue must be there to make sure that they understand why they are coming in in the first place. Are they economic? Are they running from prosecution? Refugees or asylum-seekers? Because they are not yet determined? Or are they tourists? Medical tourism or whatever? They have to understand the different groups of people coming in and out of the country based on different reasons. (I01)

Second, several mentioned the need to recognise the significant contributions that refugees and asylum seekers make to the economies of their host countries:

Also, I think the thing also is looking at that economic contribution of migrants as well. It needs to be linked in. We need to be working with Ministry of Finance and Health to demonstrate, “Look, you know, these migrants are actually stimulating the economy and bringing huge amounts for the sending or receiving countries, so you should invest a portion of that in health, which will you know stimulate development more but also protect the migrants.” (I26)

Several interviewees further emphasised that refugees and asylum seekers were not a burden and contributed in many different ways to their host countries:

They are actually not a burden. If the larger or wider general population understand the meaning of refugees, asylum-seekers, or even economic migrants, if they understand each of these definitions well, they would know that, of course, the host country is the one providing a safe haven because of some prosecution back in their country or even from poverty.... If you treat them well, they will give back to the society by building more and better roads, better land, better houses for you. I think, in a way, it is a win-win situation. (I01)

#### Strengthening health systems

Interviewees acknowledged the need to work towards strengthening health systems to incorporate refugee-inclusive policies and services that are responsive and sensitive to the rights and needs of refugees and asylum seekers. This was often presented as an opportunity in the context of the UHC agenda:

Yes. As I say, health coverage should be for every human being. It’s regardless whether you are refugee or not. So UHC should cover everybody. But then we also know that, in reality, there are certain policies that may not be feasible to cover everybody, and then we can find mechanisms to try to provide UHC in general, for everybody. (I07)

Most interviewees considered health as a basic need and therefore reported that health services should be provided to refugees and asylum seekers free of charge:

Yeah, of course, it should be included because healthcare access is like the basic needs—besides shelter, work, education. I think healthcare access is a basic need and everyone—including the migrants, asylum-seekers, and refugees—are supposed to have a universal access to basically healthcare and security. (I01)

Health insurance schemes were identified by some interviewees as the best solution to increase migrant healthcare access, but others described a combination of insufficient understanding or interest in health insurance schemes among refugees and risk aversion by insurance companies.

… most of the refugees in Malaysia, which are mainly from Myanmar, have no concept or have no health insurance concept. So to convince any refugee to purchase themselves health insurance is already a big task. The second part would be the insurance company to cover a population with different vulnerabilities. It is also a difficult negotiation, where usually the insurance company would like to have very minimal risk on healthcare coverage, where refugee groups often enough have more health risks compared to the host population. (I07)

Finally, when discussing the role of public health providers, interviewees mentioned that they should provide treatment regardless of legal status; but conversely, they must also follow ministry mandates and immigration laws of the country. Interviewees reported that this placed health workers in a difficult position when dealing with migrants of unclear status. Most healthcare professionals recommended that they should not be given an immigration role or refer migrant patients to immigration for verification of documents before providing necessary treatment.

#### Addressing the social determinants of health

Several interviewees mentioned the need for refugee populations to access educational and employment opportunities in addition to health services. The next quote highlights how experts acknowledged the importance of addressing the social determinants of health, specifically provision of job opportunities so they could contribute to the economy and society:

It ultimately boils down to the social determinants of health—just the ability to work will make a huge difference. A large proportion of the refugee population are within the productive age group and are able to contribute back to the economy and society. (I03)

Although interviewees recognised the importance of social determinants of health for refugees, they also acknowledged the difficulties in funding necessary programmes. Several interviewees suggested that governments should partner with other stakeholders to develop sustainable funding plans:

But for me, I think basic health needs, basic education needs, these are the universal needs. We should make it accessible to everybody. I mean, we can't blame the government. They may not have the resources. So the civil societies and the donors, they should come up with some sort of concrete plan to assist the government to implement budgeting. We can't blame the government because they also have limited resources. They need to cover the basic health needs to its own population. (I14)

#### Including refugees/migrants into livelihood and economic opportunities

Several interviewees expressed the need to promote social inclusion of those affected by forced migration and recognised it still remained a difficult task given the structural barriers of a social and political context that did not formally recognise refugees in many countries. They reported existing health policies as largely unresponsive to and noninclusive of refugee and displaced populations. Additionally, several interviewees highlighted the need to move away from humanitarian responses to more inclusive strategies.

So, those policies, it’s not just welfare. Of course, this is a humanitarian gesture, but I think that this we know and unfortunately, by just raising this message, there’s very little impact. So, we have to move a bit away from this when we do advocacy to the policymakers and just say that this is also for their own society—that they need to bring more inclusive strategies when it relates to health. (I21)

Others suggested the need to develop livelihoods strategies to promote social inclusion beyond the health sector and allow refugees and similar migrants to make their own decisions:

I think there's also a little bit of looking at the social determiners of health, in making sure they have access to livelihood, in being able to afford the out-of-pocket payments. So the solution would not solely come from the health sector, in terms of looking at health financing mechanisms or interventions from the health sector. So, it's a little bit of the social determiners. They have to have access to livelihood, formal livelihood that would allow them that choice and, you know, to make that decision about what they spend money on. (I09)

## Discussion

### Primary findings

Forced migration is a major political, social, and public health challenge in Southeast Asia. This qualitative study examined key challenges and health systems and policy responses in addressing the health needs of migrants affected by forced displacement in this region, primarily refugees and asylum seekers. Interviewees described the cumulative nature of health risks at each migratory phase. Perceived barriers to addressing migrants’ cumulative health needs were financial, juridico-political, and sociocultural, whereas key facilitators were the humanitarian stance taken by many health workers to treat everyone according to need as required by medical ethics and a positive national commitment to pursuing UHC. Across all countries, financial constraints both for migrants and for the programmes supporting them were identified as the main challenge in addressing the health needs of marginalised migrant populations. Host government and population biases were also major challenges. Sociocultural and language differences were also identified as significant barriers, in support of the literature [39–41]. Proposed strategies and solutions included finding regional solutions, adopting a different perspective, strengthening health systems within the UHC agenda, and taking a multisectoral approach to all the social determinants of health, including social inclusion and livelihoods. Responses require responsibility-sharing arrangements across actors and a framework whereby states can contribute in line with their capacities and receive support in accordance with their needs [7,42].

Our findings touch upon some of the key aspects discussed in a recent series of *BMJ* papers seeking to improve understanding of the complexities of delivering better health for migrants, tackle unhelpful stereotypes, and focus on the role of health in improving societal responses [43]. Legido-Quigley and colleagues suggest that migrant health should be understood in the context of the right to health and Sustainable Development Goal commitments to achieving UHC [4]. Authors also argue that the economic case whereby migrants contribute substantially to economic growth is compelling, whereas Wickramage and colleagues emphasise that toxic narratives complicate rational debates and hinder workable solutions [43]. Our interviewees similarly highlighted the need to consider migrant health in the context of UHC and promote a different perspective away from negative representations and present migrants and asylum seekers as net contributors to host economies [4,44,45].

Spitzer and colleagues argue that countries lack multisectoral action frameworks when dealing with the health needs of migrants and that rarely these responses focus on the social determinants of health and are designed to work across sectors [45]. Our interviewees also recognised the need to address the social determinants of health in Southeast Asia but were less explicit about the need for a multisectoral approach that includes social services, housing, and immigration systems, among other sectors [45]. A social-inclusion component was one of our interviewees’ key suggestions, in which it was emphasised that refugees and asylum seekers should have access to employment and education opportunities. Although our interviewees were ready to provide key solutions and recommendations, there was less insistence in recognising the need to build on individual and community strengths, promote community empowerment, and recommend participatory approaches [45]. Only one interviewee referred briefly to such needs.

Veary and colleagues highlighted that migrant healthcare cannot be isolated from historical, sociopolitical, economic, and legal contexts [44]. Our research similarly highlighted that contextual factors are important when studying forced migration with any policy aiming to improve the well-being of refugees and other migrants needing to consider the situational, structural, cultural, and exogenous factors specific to the region. For example, in Southeast Asia, situational factors included the sudden escalation of cross-border mass forced displacement. Major structural factors included that most countries were non-signatories to the 1951 Refugee Convention and, hence, did not accord refugees legal status. Sociocultural factors included differences in perceptions and experiences between migrant and host populations that deterred integration. Exogenous factors included regional cooperation and responsibility sharing as essential elements for developing solutions because of the cross-border nature of migratory movements.

Veary and colleagues further identified the need to develop governance responses to migration and health and suggested a “migration and health in all policies” approach [44]. Authors stressed the importance of building alliances across sectors to support migration governance responses but acknowledged that governance of migration and health is primarily an issue of state sovereignty [44]. Our interviewees highlighted that effective governance was needed and all actors had a role in finding solutions, including UN agencies, health and other relevant ministries, international and national NGOs and community-based organisations, pressure/interest groups, media, private-sector organisations, health facilities and health workers, funding agencies, and governments. At the same time, interviewees also agreed that governments in the region had enduring dominance over other actors in the formulation and evolution of migrant-inclusive health policies and countries still had a long way to go to provide ethical evidence-/justice-driven policy responses.

### Strengths and limitations

To our knowledge, this is one of the first qualitative studies of which we are aware to investigate the health concerns and barriers to access among refugees and asylum seekers in several countries in Southeast Asia. Findings provide new and useful insights with practical implications for informing policy and interventions directed at addressing the health needs of refugees and others experiencing forced displacement. A strength of this study was the data collection process of capturing the viewpoints of a wide range of actors from different sectors, and use of semistructured interviews allowed for a comprehensive exploration of emerging issues.

Given study objectives, to explore migrant healthcare needs and access, we would ideally conduct interviews with refugees and asylum seekers to better understand their perspectives. However, our choice of healthcare professionals, programme staff, and refugee health experts as interviewees was partly to determine the feasibility and appropriateness of interviewing potentially vulnerable refugees and asylum seekers in future research, given such research would add value to the literature and help corroborate study findings. Nonetheless, interviewing healthcare professionals, programme staff, and refugee health experts provided a unique perspective on this topic, teasing out broader issues and gaps in health systems and service delivery. This interview sample were individuals with a keen interest in the topic who were very outspoken in sharing their views. Collectively, this may have limited the breadth of perspectives and resulted in potential biases whereby interviewees recruited were largely sympathetic to refugees. Nevertheless, we observed that most interviewees maintained objectivity in their views by providing a holistic presentation of the issues discussed.

### Implications for policy and practice

In many Southeast Asian countries, the social inclusion of refugee populations and those affected by forced migration remains a difficult task given systemic barriers caused by sociopolitical structures that do not formally recognise refugees and asylum seekers. Interviewees expressed the need for a different perspective in responding to these often vulnerable populations, who have managed to become significant contributors to host-country economies. The public health benefits of refugee recognition and migrant-inclusive approaches are significant and extend beyond preventing disease spread to improving social equity, designing livelihood frameworks, and migrant and host population wellness. Implementation of health insurance schemes for migrants could be a way to improve access for different populations. Using regional platforms, such as ASEAN, more effectively could facilitate discourse on resolving root causes and addressing the health-related issues of forced migration. Interviewees focused on regional-level responses, but closer engagement with commitments made by the Global Compact on Migrants and Refugees and the Global Action Plan on Migrants and Refugees would also strengthen government responses and better align them with global approaches. Overall, in line with a people-centred and inclusive post-2015 global health agenda, interviewees acknowledged the need to work towards strengthening health systems that incorporate migrant-inclusive policies and services that are responsive, financially sustainable, and sensitive to the rights and needs of refugees and asylum seekers.

### Conclusions

This is one of the first qualitative studies of which we are aware to investigate the health concerns and barriers to access among migrants and refugees experiencing forced displacement in the context of health system challenges and responses to the Andaman Sea refugee crisis in Southeast Asia. Findings provide useful new insights with practical implications for informing policy and interventions. Strategies proposed by participants, which could be considered, include adopting a ‘migration and health in all policies’ approach; developing regional solutions recognising the crucial role of national governments; adopting a different perspective in which refugee and asylum-seeker contributions are recognised and valued in host societies; adopting a multisectoral approach to strengthening health systems within the Sustainable Developments Goals and UHC agendas; and addressing the social determinants of health of both migrant and host populations by promoting social-inclusion and livelihoods frameworks.

## Supporting information

S1 COREQ Checklist (PDF)

S1 Interview Guide (DOCX)

## Abbreviations

ASEAN: Association of Southeast Asian Nations
CSO: civil-society organisation
GO: governmental organisation
ICRMW: International Convention on the Protection of the Rights of All Migrant Workers and Members of Their Families
IDP: internally displaced person
IOM: UN International Organization for Migration
JKN: Jaminan Kesehatan Nasional
NGO: nongovernmental organisation
TB: tuberculosis
UHC: universal health coverage
UNHCR: UN High Commissioner for Refugees
