# Supplementary material for: Southeast Asian health system challenges and responses to the ‘Andaman Sea refugee crisis’: A qualitative study of health-sector perspectives from Indonesia, Malaysia, Myanmar, and Thailand
Source: PLoS Med. 2020 Nov 10;17(11):e1003143. doi: 10.1371/journal.pmed.1003143 (PMC7654775; doi:10.1371/journal.pmed.1003143)
Supplement: S1 Interview Guide — (DOCX) [file pmed.1003143.s002.docx]

#
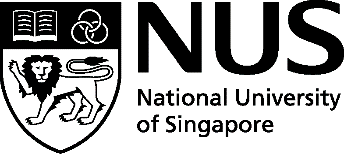
TOPIC GUIDE:

# KEY INFORMANT INTERVIEWS

***PROJECT TITLE***

***Health Systems Responses and Challenges of the Refugee Crisis in South-East Asia***

**Pre-interview**

Hello and thank you for agreeing to be interviewed today for our research study that aims to investigate how health systems in South-east Asian respond to crises of forced displacement and deal with refugee health issues. Through our study, we hope to gain a better understanding of the health needs and challenges of refugee and asylum-seeker population through the lens of policy-makers and experts in health and migration; the barriers and challenges faced by health systems in addressing these needs and to explore how health systems can respond more effectively to crises of forced displacement in the region.

The interview will be audio recorded and will go on for about an hour. Please let me know if it is alright for me to record you. (Pause for response). Also, please let me know if you would like me to stop the recording at any time. In any event that you feel uncomfortable answering any of the questions I ask, you can let me know and I will move on to the next question. Before I start, do you have any questions for me regarding the research project that this interview is for, the interview procedures, or anything else?

(Respond to questions, if any.) I will now turn on the audio recording device, and we can start.

**Introductory Question**

1. Can you tell me about your professional experience or the work that you have done in the area of refugee and migrant health over the course of your career?

**Actors**

1. Who are the main actors involved in dealing with refugee health in (insert country)?
2. What are the roles of each of the actors mentioned?
3. How is the relationship between the various actors in terms of coordination and collaboration? How do the various actors work together on refugee health issues?
4. Over the years, in what ways have the government been involved in refugee health issues? What has been your experience working with the government on refugee health issues? How better to engage government stakeholders on issues relating to refugee health?

**Context, Social Determinants of Health and Health Needs**

1. Can you describe the refugee situation in (insert country)?
2. How is it like for asylum seekers and refugees to live in (insert country)? (prompt: access to employment, education, housing and other social schemes)
3. What are the most common health problems among asylum seekers and refugees in (insert country)?
4. How do refugees and asylum seekers access public health services in (insert country)?
5. What are the barriers that refugees and asylum seekers face in accessing health services? (prompt: protection barriers, financial barriers, structural barriers, language barriers, cultural barriers, health literacy barriers)

**Institutional Frameworks and Legal Policies**

1. Most countries in the region lack formal mechanisms or legal frameworks to process refugees and asylum seekers, what implications does this have on the health of refugees and asylum seekers?
2. What are some of the national immigration policies that have direct implications on the health of refugees and asylum seekers in (insert country)?
3. To what extent is refugee health a priority in the policy-making arena within the health sector?
4. To what extent are national health policies ‘migrant-inclusive’ in (insert country)? How would you define ‘migrant-inclusive’ health services?
5. What are the national health policies and plans that are directly related to asylum seekers and refugees in (insert country)? What are the latest policy developments? What implications have these developments brought on to the health of asylum seekers and refugees?
6. What do you think are some factors that make advocating for ‘migrant-inclusive’ policies challenging? How can these challenges be addressed?
7. What do you think are some of the factors that could facilitate the implementation of refugee-friendly health policies?
8. Countries in the region have made progress towards attaining Universal Health Coverage (UHC) for its citizens; to what extent are the refugee and asylum seeker population included in the provision of UHC?

**Health Systems Responses**

*Broader questions on health system responses to refugee health problems*

1. What are the implications of having large populations of asylum seekers and refugees on the health system of (insert country)?
2. What are the main challenges faced by health systems in the delivery of healthcare services to the asylum seeker and refugee population?
3. In your opinion, do health systems have the capacity in extending services to the asylum seeker and refugee population? (prompt: in terms of healthcare workforce, and availability of medical goods and services)
4. What is the degree of coordination and collaboration between the Ministry of Health and other stakeholders (particularly UNHCR) in addressing refugee health issues? (prompt: in terms of information sharing, coordination of care, resolving ad-hoc refugee health issues?
5. What are the current health care financing sources for refugees in (insert country)? (prompt: insurance schemes, out-of-pocket payment, subsidies). What is your opinion on the current national healthcare financing structures for the asylum seeker and refugee population?
6. Do you know of any health financing schemes or mechanisms that could be effective in improving an asylum seeker or refugee’s access to healthcare?

*Specific questions (adapt to each country)*

1. What are your key concerns on the migratory crisis?
2. How did governments in the region respond to the health needs of the refugees throughout the crisis? What are your views on the way they responded?
3. What implications did this crisis have on the health systems of countries most affected by the influx of asylum seekers?
4. During the crises, who were the main actors involved in responding to the health needs of the refugees? How was the response coordinated? What was the level collaboration between the actors in responding to the crisis?
5. In your opinion, what is the role of national health systems in responding to such crises?
6. In your opinion, what is the role of regional networks and institutions in responding to such crises?
7. The 2015 boat people crisis brought about some regional efforts in responding to the crisis through regional meetings and forums like the Bali Process, in your view, how effective were these efforts in responding to the health needs of the boat refugees affected by the crisis?
8. In your opinion, how well was the overall response at national and regional levels in dealing with the health risks of those affected by the crisis?
9. In your opinion, for countries not affected by the crisis, do they play a role in the overall regional response to such crisis?
10. In your opinion, how could the various actors have responded more effectively in addressing the health risks of those affected by the crisis in the different phases throughout the migratory process? (prompt: pre-departure, travel, interception and arrival phase)
11. Can you tell us about your experience in your own country?
12. What are the main challenges?
13. What could be done to improve the situation?

*Specific questions relating to the Bay of Bengal and Andaman Sea boat people crisis in 2015*

1. In 2015, there was a refugee boat people crisis in the Bay of Bengal and the Andaman Sea. From your recollection, what were some of the most pressing health needs among those undertaking the boat journeys?
2. How did governments in the region respond to the health needs of the refugees throughout the crisis? What are your views on the way they responded?
3. What implications did this crisis have on the health systems of countries most affected by the influx of asylum seekers?
4. During the crises, who were the main actors involved in responding to the health needs of the refugees? How was the response coordinated? What was the level collaboration between the actors in responding to the crisis?
5. In your opinion, what is the role of national health systems in responding to such crises?
6. In your opinion, what is the role of regional networks and institutions in responding to such crises?
7. The 2015 boat people crisis brought about some regional efforts in responding to the crisis through regional meetings and forums like the Bali Process, in your view, how effective were these efforts in responding to the health needs of the boat refugees affected by the crisis?
8. In your opinion, how well was the overall response at national and regional levels in dealing with the health risks of those affected by the crisis?
9. In your opinion, for countries not affected by the crisis, do they play a role in the overall regional response to such crisis?
10. In your opinion, how could the various actors have responded more effectively in addressing the health risks of those affected by the crisis in the different phases throughout the migratory process? (prompt: pre-departure, travel, interception and arrival phase)

**Closing Questions**

1. Before we wrap up, do you have any final thoughts that you would like to share?
2. This brings us to end of our interview. Do you have any final questions?
